# Evaluation of 2-[^18^F]-Fluorodeoxysorbitol PET Imaging in Preclinical Models of *Aspergillus* Infection

**DOI:** 10.3390/jof8010025

**Published:** 2021-12-28

**Authors:** Jianhao Lai, Swati Shah, Rekeya Knight, Neysha Martinez-Orengo, Reema Patel, Amelia Mitchell, Zeping Wang, Falguni Basuli, Alvaro A. Ordonez, Sanjay K. Jain, Dima A. Hammoud

**Affiliations:** 1 Center for Infectious Disease Imaging, Radiology and Imaging Sciences, National Institutes of Health, Bethesda, MD 20892, USA; Jianhao.lai@nih.gov (J.L.); swati.shah@nih.gov (S.S.); rekeyak@gmail.com (R.K.); neysha.martinez-orengo@nih.gov (N.M.-O.); rp5mg@virginia.edu (R.P.); amelia.mitchell325@gmail.com (A.M.); zptwang96@gmail.com (Z.W.); 2 Chemistry and Synthesis Center, National Heart, Lung, and Blood Institute, National Institutes of Health, Rockville, MD 20892, USA; bhattacharyyaf@nhlbi.nih.gov; 3 Department of Pediatrics, The Johns Hopkins University, Baltimore, MD 21287, USA; aordone2@jhmi.edu (A.A.O.); sjain5@jhmi.edu (S.K.J.)

**Keywords:** PET imaging, *Aspergillus* infection, FDS, FDG

## Abstract

Despite increasing associated mortality and morbidity, the diagnosis of fungal infections, especially with *Aspergillus fumigatus* (*A. fumigatus*), remains challenging. Based on known ability of *Aspergillus* species to utilize sorbitol, we evaluated 2-[^18^F]-fluorodeoxysorbitol (FDS), a recently described *Enterobacterales* imaging ligand, in animal models of *A. fumigatus* infection, in comparison with 2-[^18^F]-fluorodeoxyglucose (FDG). In vitro assays showed slightly higher ^3^H-sorbitol uptake by live compared with heat-killed *A. fumigatus*. However, this was 10.6-fold lower than *E. coli* uptake. FDS positron emission tomography (PET) imaging of *A. fumigatus* pneumonia showed low uptake in infected lungs compared with FDG (0.290 ± 0.030 vs. 8.416 ± 0.964 %ID/mL). This uptake was higher than controls (0.098 ± 0.008 %ID/mL) and minimally higher than lung inflammation (0.167 ± 0.007 %ID/mL). In the myositis models, FDS uptake was highest in live *E. coli* infections. Uptake was low in *A. fumigatus* myositis model and only slightly higher in live compared with the heat-killed side. In conclusion, we found low uptake of ^3^H-sorbitol and FDS by *A. fumigatus* cultures and infection models compared with *E. coli*, likely due to the need for induction of sorbitol dehydrogenase by sorbitol. Our findings do not support FDS as an *Aspergillus* imaging agent. At this point, FDS remains more selective for imaging Gram-negative *Enterobacterales*.

## 1. Introduction

*Aspergillus fumigatus* (*A. fumigatus*) is an opportunistic pathogen for immunocompromised subjects. Although immunocompetent subjects may inhale up to 1000 conidia each day, those are usually cleared by lung epithelial cells and macrophages [1]. In immunocompromised patients such as those undergoing chemotherapy or organ transplants, on the other hand, fungal conidia can evade the immune system, germinate, and invade lung tissues, resulting in severe and often fatal invasive pulmonary aspergillosis (IPA) [2,3,4]. *A. fumigatus* affects over 200,000 patients worldwide, with mortality rates between 30% and 90% [5]. IPA, for one, has emerged as the leading cause of infectious death in immunocompromised patients [6]. Due to its prevalence and costly treatments, IPA is also the most expensive fungal disease [7].

One reason for the high lethality rates of fungal infections, especially *A. fumigatus*, is the low sensitivity and specificity of available diagnostic methods. Standard imaging procedures such as computed tomography (CT) scans, cultures of sputum/bronchoalveolar lavage (BAL) samples, or galactomannan tests are currently available but are slow to provide results and often have limited sensitivity and specificity [8,9,10,11]. Most of those tests also require collection of blood or tissue samples, which can be invasive, and may not accurately represent the local biology at infection sites [12]. Therefore, new non-invasive methods to diagnose the early onset of fungal infections, including IPA, are urgently needed. Molecular imaging techniques such as single-photon emission computed tomography (SPECT) or positron emission tomography (PET) surpass structural imaging by their ability to provide functional and/or molecular information [13]. Among those, ^18^F-fluorodeoxyglucose (FDG) can be useful for the evaluation of invasive fungal infection, but it still cannot distinguish between infectious etiologies, cancer, and inflammation [14]. Over the last couple of decades, multiple imaging probes have been designed and tested for specific diagnosis of fungal diseases, including radiolabeled siderophores [3,15,16], phosphorodiamidate morpholino (MORF) oligomers [17], and radio-labeled antibodies [18,19,20]. Although very promising, none of those ligands have made it into the clinic yet.

Sorbitol, a sugar alcohol obtained by reduction of glucose, is a metabolic substrate for *Enterobacterales* and is not metabolized by mammalian cells [21]. The positron-emitting analog of sorbitol, 2-[^18^F]-fluorodeoxysorbitol (FDS), first described by Li et al. for tumor imaging [22], was later proven to be a suitable selective in vivo probe in preclinical models of *Enterobacterales* infections [23]. With high kidney extraction and excretion, low plasma protein binding, and high metabolic stability, FDS was also found to be useful for measuring renal function [24]. The safety, biodistribution, and radiation dosimetry of FDS have already been evaluated in humans [25] and a recent study proved its usefulness in diagnosing *Enterobacterales* infections in patients [26].

The basis for using FDS to diagnose fungal infections is the known sorbitol metabolism by certain *Aspergillus* species. The presence of sorbitol was found to induce the expression of sorbitol dehydrogenase in *Aspergillus niger* and upregulate the corresponding gene, *sdhA*, which is essential for sorbitol catabolism [27]. In our study, we synthesized FDS from commercial FDG. The in vitro uptake assays of ^3^H-sorbitol and FDS were performed in different fungal and bacterial species, along with the PET imaging studies of FDS and FDG in animal models of *A. fumigatus* pneumonia and fungal/bacterial myositis. Our goal was to establish the potential utility of FDS in diagnosing fungal infections.

## 2. Materials and Methods

### 2.1. Synthesis of FDS

All chemicals used in the study were purchased from Sigma-Aldrich (St. Louis, MO, USA) and used without further purification. The Sep-Pak^®^ cartridges were obtained from Waters (Milford, MA, USA). FDG was purchased from Cardinal Health (Beltsville, MD, USA). FDS was prepared according to the literature method with minor modifications [28]. Briefly, FDG (~2 mL saline) was reduced with sodium borohydride (13 mg in 0.3 mL water) at 60 °C for 5 min followed by quenching with a mixture of 1 N HCl (0.3 mL) and 1.25 N NaOAc (0.7 mL). The reaction mixture was passed through an activated (6 mL saline) Sep-Pak^®^ plus Alum-N and Millex-GS (0.2 µm) sterile filter to collect the eluent in a vial. The system was flushed with 4 mL saline and the eluent was collected in the same vial to obtain FDS. The overall radiochemical yields were 59–71% (decay-uncorrected, *n* = 15) in 20 min synthesis time. A radiochemical purity of >99% was determined by high-performance liquid chromatography (HPLC).

### 2.2. Bacterial and Fungal Strains

*Aspergillus fumigatus* B-5233 clinical isolate was obtained from a case of invasive aspergillosis at the National Institutes of Health (NIH) (kindly donated by Dr. K.J. Kwon-Chung from the National Institute of Allergy and Infectious Diseases (NIAID), NIH, Bethesda, MD, USA). *Candida albicans* (*C. albicans*, strain SC5314) was kindly donated by Dr. Michail Lionakis from NIAID, NIH. Representative bacterial strains of *Pseudomonas aeruginosa* (*P. aeruginosa*) were isolated from Chronic Granulomatous Disease and Autosomal Dominant Hyper-IgE Syndrome patients from the Department of Microbiology at the Clinical Center of the National Institutes of Health, Bethesda, MD, USA [29]. *Aspergillus niger* (*A. niger*, strain ATCC 1015), *Escherichia coli* (*E. coli*, strain ATCC 25922), *Staphylococcus aureus* (*S. aureus*, strain 29213), and J774A.1 murine macrophage cell line (strain TIB-67) were purchased from American Type Culture Collection (ATCC, Manassas, VA, USA).

### 2.3. In Vitro Uptake Assay

#### 2.3.1. Bacterial, Fungal, and Cell Cultures

Bacterial strains (*E. coli*, *S. aureus*, and *P. aeruginosa*) were grown in Lysogeny Broth (LB) media overnight at 37 °C with shaking. Bacterial culture pellets were resuspended in fresh LB media and were adjusted to an optical density of 1.0 at 600 nm (OD_600_) before use. *A. fumigatus* and *A. niger* hyphae were grown in yeast glucose (YG) media at a concentration of 1 × 10^5^ conidia/25 mL (FDS and FDG uptake assay) or 1 × 10^4^ conidia/30 mL (^3^H-sorbitol and ^3^H-2-deoxyglucose (^3^H-2-DG) uptake assay), washed once with phosphate-buffered saline (PBS), and resuspended in minimal media (6 g/L NaNO_3_, 0.52 g/L KCl, 0.52 g/L MgSO_4_·7H_2_O, 1.52 g/L KH_2_PO_4_, adjusted pH to 6.5, 2 g/L glucose, 2 mL/L Hunter’s trace elements) as an approach to simulate normal blood glucose levels in humans. *C. albicans* was grown in yeast extract–peptone–dextrose (YPD) media overnight at 30 °C with shaking. Before the assay, *C. albicans* was resuspended in minimal media to a concentration of 1 × 10^8^ cells/2.5 mL. One day before the uptake assay, J774 murine macrophages were seeded in a concentration of 1.5 million/well (in 6-well plates) in a final volume of 2.5 mL/well low glucose growth media (DMEM 1 g/L D-glucose, Gibco, Gaithersburg, MD, USA) and incubated at 37 °C in a humidified atmosphere containing 5% CO_2_.

#### 2.3.2. ^3^H-Sorbitol and ^3^H-2-DG Uptake Assays

Bacterial and fungal (hyphae) cultures were incubated, in triplicates, with the radiolabeled sugar (^3^H-sorbitol or ^3^H-2-DG, American Radiolabeled Chemicals, St. Louis, MO, USA; 0.5 μCi/2.5 mL culture) at 37 °C with shaking at 200 rpm and samples were collected after 30, 60, and 120 min. Heat-killed bacterial (95 °C for 30 min in *E. coli* and *P. aeruginosa*; 60 min in *S. aureus*) and fungal (70 °C for 60 min) cultures underwent the same incubation conditions with the radiolabeled sugars, collected at the same time points (in duplicates), and used as negative controls.

For comparing conidial and hyphal stages of uptake, a concentration of 1 × 10^7^ *A. fumigatus* conidia/mL in a total volume of 2.5 mL minimal media was used in triplicates and incubated with the radiolabeled sugar following the same procedure described above.

For J774 cells, supernatants were discarded and replenished with no glucose media (DMEM without glucose). Radiolabeled sugars were added at the same concentration as the bacterial and fungal cultures, cells were placed in the incubator, and samples were collected at 10, 30, and 120 min. All samples were pelleted by centrifugation with intercalated washes of PBS (×3).

For sorbitol pre-incubation study, *A. fumigatus* and *E. coli* were cultured overnight as previously described. On the day of the experiment, *A. fumigatus* fungal culture was filter-washed once with PBS and resuspended in either minimal media (2 g/L glucose) or minimal media containing sorbitol (no glucose, 10 g/L sorbitol). *E. coli* pellets were resuspended in fresh LB media. Cultures were incubated for 2 h with shaking at 37 °C. After incubation, *A. fumigatus* was washed once with PBS and resuspended in minimal media containing glucose. *E. coli* was centrifuged and resuspended in LB media. ^3^H-sorbitol uptake assay was performed as previously described.

Pellet dry weights of bacteria and *C. albicans* were obtained after the washes. Due to the morphological characteristics of *Aspergillus* hyphal structures, it was difficult to remove all the supernatant through aspiration alone. Thus, those pellets were treated with 4% paraformaldehyde (PFA), placed on filter paper, and allowed to dry at room temperature overnight before recording their final weights.

In the case of the conidia, we used tubes fitted with 0.22 µm filters (Fisher Scientific, Waltham, MA, USA) for sample collection and washes. Pellets were resuspended in 1 mL scintillation fluid (Ultima Gold XR, PerkinElmer, Chicago, IL, USA) and left at room temperature. After 24 h, the associated radioactivity was measured using a MicroBeta2 counter (PerkinElmer).

Counts for bacterial, *C. albicans*, and *Aspergillus* hyphal assays were normalized to pellet weight and ^3^H-2-DG uptake. Fungal hyphal assays that were compared with conidial assays were normalized to 10^7^ conidia.

#### 2.3.3. FDS and FDG Uptake Assays

*E. coli* and *A. fumigatus* cultures were incubated with FDG or FDS (0.5 μCi/mL culture) at 37 °C with shaking at 200 rpm. After 30, 60, and 120 min of incubation, 1 mL samples were collected in triplicates. Heat-killed bacterial (95 °C for 30 min) and fungal (70 °C for 60 min) cultures underwent the same incubation conditions, were collected at the same time points (in duplicates) and used as negative controls. Samples underwent three PBS washes. Activity for each sample pellet was measured immediately using an automated gamma counter (PerkinElmer). Counts were corrected for background and decay and normalized to pellet weight.

### 2.4. Animal Infection

All experimental procedures, including handling and care of the animals, were performed in an AAALAC International accredited facility in accordance with relevant NIH policies and the Animal Welfare Act and Regulations and were approved by the Animal Care and Use Committee of the Clinical Center of the NIH.

Female CD-1 mice (6 to 7 weeks old, Charles River, Charleston, SC, USA) were used for pulmonary *Aspergillus* infection and lung inflammation models, and female BALB/c mice (6 to 8 weeks old, Charles River, Charleston, SC, USA) were used for myositis models. All mice were housed in pre-sterilized filter-topped cages and given access to food and water ad libitum.

Live pathogen: *A. fumigatus* was initially grown on Malt extract agar (MEA) slants for 3–5 days at 37 °C. The conidia were collected in sterile PBS with 0.1% Tween 20 (PBST) and passed through 40-µm nylon filters. The conidia were washed twice, counted on a hemocytometer, and finally resuspended in PBST.

Heat killing: *Aspergillus* conidia were grown for 3–5 h in rich nutrient media to allow for conidial swelling and germination. At this stage, the suspension was autoclaved at 121 °C for 30 min to ensure the complete killing of any residual conidia.

#### 2.4.1. Lung Infection Model

To induce immunosuppression, cyclophosphamide was injected intraperitoneally (IP) 4 days (150 mg/kg) and 1 day (100 mg/kg) before inoculation to render the mice neutropenic. Pulmonary aspergillosis was then induced in the immunosuppressed mice by inoculating the mice via the post-pharyngeal aspiration (PPA) technique as described previously [30,31]. Briefly, while under anesthesia, the animal was suspended by its top incisors on a suspension stand and the tongue was fully extended using padded forceps. A 30 µL suspension of fungal conidia (0.5–1 × 10^7^ CFU/mouse) was then pipetted onto the base of the tongue and the restraint was maintained until at least ten breaths are completed. These mice were imaged 2 days post infection. The dose and timing of imaging were selected based on the establishment of an identifiable infection in the lung (detected by lung CT and Grocotts Methenamine Silver (GMS) staining) without progressing into sepsis with kidney failure or death.

#### 2.4.2. Intravenously Infected Model

A 100 µL *A. fumigatus* conidial suspension (0.5 to 1 × 10^7^ CFU/mouse) was injected into tail veins of immunocompetent CD-1 mice 2 days before imaging to induce brain infection [32].

#### 2.4.3. Lung Inflammation Model

To test the specificity of the radiolabeled sugars, a lung inflammation model was developed by inoculation of female CD-1 mice with polyinosinic–polycytidylic acid (poly(I:C)) using a post-pharyngeal approach. While under anesthesia, a 25 µL suspension containing 200 µg of poly(I:C) was given for a total of 3 times with 24 h intervals between them. The animals were imaged 24 h after the last treatment.

#### 2.4.4. Aspergillus Myositis Model

A 100 µL suspension containing 5 × 10^7^ live (right thigh) and 5 × 10^7^ heat-killed *A. fumigatus* (left thigh) were injected intramuscularly (IM) into the immunosuppressed mice thighs on the right and left sides, respectively. Live conidia were injected 3 days before imaging, while the heat-killed solution was injected 20 h before imaging to avoid clearance by the immune system.

#### 2.4.5. Bacterial Myositis Model

Immunocompetent female BALB/c mice were used as *E. coli* and *S. aureus* infection models since this mouse background provided the most reliable and reproducible myositis model in our hands. The mice were injected IM with a 100 µL suspension containing 1 × 10^8^ live *S. aureus* or 5 × 10^8^ live *E. coli* in the right thigh and 1 × 10^10^ heat-killed *E. coli* or *S. aureus* in the left thigh. For heat-killing, the bacterial cultures were incubated at 95 °C for 1 h. Heat killing was verified periodically through culturing. PET imaging was performed 4 to 6 h after inoculation with *E. coli* or *S. aureus*. The animals were euthanized following the scans and their thigh muscles were collected.

### 2.5. PET/CT Imaging

For the in vivo PET imaging experiments, we used either the nanoScan PET/CT (Mediso, Budapest, Hungary) or the Inveon small-animal PET/CT scanner (Siemens Medical, Knoxville, TN, USA). Images of all the myositis models were collected by nanoScan PET/CT, while the pulmonary infection, lung inflammation animal models, and corresponding healthy controls were scanned using the Inveon PET/CT. Mice were imaged after injection of 0.1 mL FDS or FDG solution (~9.25 MBq) via the tail vein. The mice were anesthetized with isoflurane and placed on a heating pad to keep them warm throughout scanning. 15 min static PET images were acquired at 30 min after intravenous injection of FDG or 120 min after injection of FDS, followed by CT. A subset of the bacterial myositis model animals was scanned at both 30 and 120 min after injection of FDG to assess the difference in FDG PET between the two time points. For dynamic imaging with FDS, radioactivity was injected 10 s after the emission scan was started. Data acquisition continued for 45 min.

For both static and dynamic scans, regions of interest (ROIs) were drawn over the tissues of interest based on the anatomical information from the CT images and analyzed using PMOD software (PMOD Technologies Ltd., Zürich, Switzerland) or Fusion software (Mediso Ltd., Budapest, Hungary). The same sets of images were analyzed by two separate operators and results were compared and solidified into one data set. Uptake is expressed as %injected dose/mL (%ID/mL). Uptake in intramuscular infection sites and normal muscle were quantified to calculate the target to non-target (T/NT) ratio. Time–activity curves (TAC) were generated from the dynamic scans.

### 2.6. GMS Staining

After PET imaging, mice were transcardially perfused with normal saline and 4% PFA prior to tissue collection. The lungs and/or brain were embedded in OCT (Tissue-Tek) and stored at −80 °C until they were ready to be sectioned. Thigh muscles from myositis mice were removed and soaked in 4% PFA before embedding in the OCT. Following this, 10–15 µm sections were stained with GMS (ScyTek Laboratories Inc., West Logan, UT, USA) to identify fungal hyphae. Images of stained sections were collected using Aperio ScanScope (Leica Biosystems, Buffalo Grove, IL, USA) or Eclipse E200 (Nikon, Melville, NY, USA).

### 2.7. Statistical Analyses

GraphPad Prism 8 software (GraphPad Software, San Diego, CA, USA) was used for statistical analyses. Statistical significance was determined by paired or unpaired two-tailed *t*-test based on the experimental design (longitudinal versus cross-sectional). A *p* value < 0.05 was considered statistically significant. Quantitative data are expressed as mean ± standard error of the mean (SEM).

## 3. Results

### 3.1. In Vitro Uptake Assay

**Figure 1:**
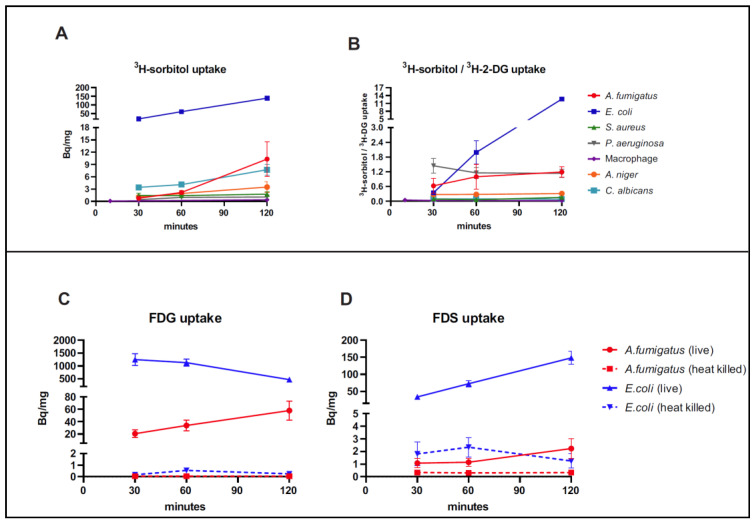
In vitro uptake assays. (**A**) ^3^H-sorbitol uptake and (**B**) ratio of ^3^H-sorbitol uptake to ^3^H-2-Deoxyglucose (DG) uptake in *A. fumigatus*, *E. coli*, *S. aureus*, *P. aeruginosa*, *A. niger*, *C. albicans*, and J774A macrophages. (**C**) 2-[^18^F]-fluorodeoxysorbitol (FDS) uptake and (**D**) ^18^F-fluorodeoxyglucose (FDG) uptake in live and heat-killed *E. coli* and *A. fumigatus* cultures, incubated for 120 min. Heat-killed *A. fumigatus* or *E. coli* were used as negative controls. Data are expressed as means ± SEM of 3 or more replicate experiments.

In the uptake assays using ^3^H-sorbitol and ^3^H-2-DG, *E. coli* had the highest rate of accumulation of ^3^H-sorbitol at all time points while *A. fumigatus* had moderate uptake (when normalized to weight) (Figure 1A). The uptake in *A. fumigatus* was higher than that seen in J774 macrophages, *S. aureus*, and *P. aeruginosa*, which have been previously described as having low FDS uptake [23]. The ^3^H-sorbitol uptake was similar in *A. niger* and *A. fumigatus* in the first 60 min post incubation but increased in *A. fumigatus* by 120 min. The uptake in *C. albicans* was slightly higher than in *A. fumigatus* when normalized to weight from 30 min to 60 min however it was lower than *A. fumigatus* at 120 min (Figure 1A).

When we compared the ratio of ^3^H-sorbitol retention to ^3^H-2-DG retention in the evaluated pathogens (Figure 1B), *E. coli* showed the highest ratio, far higher than all other pathogens, consistent with preferential uptake of sorbitol compared with 2-DG. This was most notable at 120 min. Meanwhile, *A. fumigatus* showed a higher ratio than J774 macrophage, *S. aureus*, and *P. aeruginosa*. However, *A. fumigatus* uptake was much lower than *E. coli* uptake (13.4-fold lower) when normalized to weight and 10.6-fold lower when normalized to ^3^H-2-DG uptake.

We also incubated live and heat-killed *A. fumigatus* and *E. coli* with FDS or FDG. We found that at 120 min, more FDS was retained by live *A. fumigatus* compared with heat-killed *A. fumigatus* (6.75-fold higher), but the uptake was still lower than that of live *E. coli* (66-fold lower) when normalized to weight (Figure 1C). Both *E. coli* and *A. fumigatus* had higher uptake of FDG than that of FDS, with very low uptake seen in the corresponding heat-killed cultures (Figure 1D).

The uptake of ^3^H-sorbitol and ^3^H-2-DG in *A. fumigatus* conidia was insignificant compared with uptake in the hyphal stages of growth (up to two hours post incubation) (Figure S1).

When comparing *A. fumigatus* cultures grown on sorbitol-rich (10 g/L sorbitol) versus minimal media, there was marked upregulation of ^3^H-sorbitol uptake in the presence of sorbitol (~23 fold at 120 min) consistent with *SdhA* upregulation as has been previously described [27] (Figure S2).

### 3.2. Dynamic PET Imaging with FDS in Control and Infected Models

**Figure 2:**
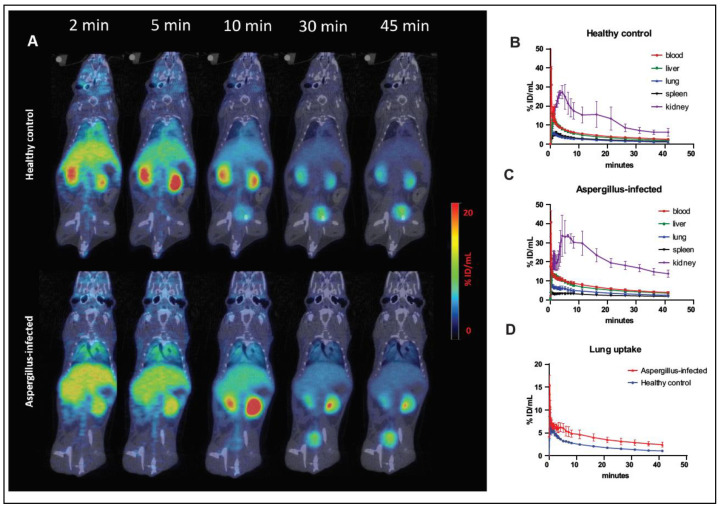
Dynamic PET/CT imaging with 2-[^18^F]-fluorodeoxysorbitol (FDS) in mice with *A. fumigatus* pulmonary infection and healthy controls. (**A**) Representative serial coronal sectional PET/CT images are shown. (**B**) FDS time–activity curves of various organs in control animals. (**C**) FDS time–activity curves of various organs in *Aspergillus*-infected animals. (**D**) FDS time–activity curves of lungs in control animals and *Aspergillus*-infected animals. Data are expressed as means ± SEM. (%ID/mL: %Injected dose/mL).

Dynamic PET images (Figure 2A) in control animals showed rapid renal excretion of FDS. There was some initial uptake in the liver, but the PET signal decreased significantly by 45 min post injection. Time–activity curves (Figure 2B) showed that FDS has a short blood half-life in healthy animals (about 6 min) and the highest uptake was in the kidneys. Dynamic PET scans in *Aspergillus* pneumonia models showed similar distribution and excretion of FDS as healthy animals, although the blood half-life was longer in infected animals (about 10.8 min) (Figure 2C). Moreover, the uptake in the lungs of infected animals was higher than that of control animals (Figure 2D).

### 3.3. Static Delayed FDS and FDG PET Imaging in Control, Infected, and Lung Inflammation Models

**Figure 3:**
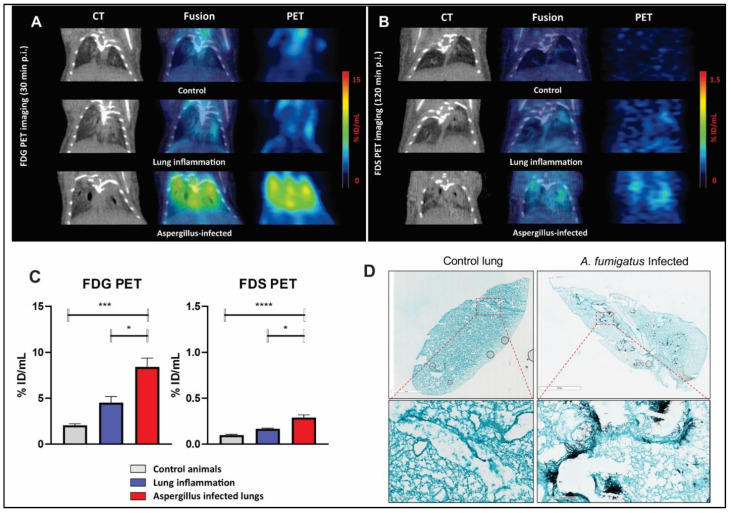
Static PET/CT imaging with ^18^F-fluorodeoxyglucose (FDG) (30 min post injection) or 2-[^18^F]-fluorodeoxysorbitol (FDS) (120 min post injection) in *A. fumigatus* infected mice compared with healthy controls and poly(I:C)-induced lung inflammation models. (**A**,**B**) Representative coronal sectional images of lung CT, PET, and PET/CT fusion are shown. (**C**) Quantified uptake of FDG or FDS in the lungs of control, lung inflammation, or *A. fumigatus* infected mice. (**D**) GMS staining of lung slices from a control mouse and an *A. fumigatus* infected mouse on day 3 after infection. Scale bars, 2 mm (upper row panels) and 200 µm (lower row panels). Data are expressed as means ± SEM. *, *p* < 0.05. ***, *p* < 0.001. ****, *p* < 0.0001. (%ID/mL: %Injected dose/mL).

Static PET imaging was performed at 120 min after injection of FDS and 30 min after injection of FDG to allow for the decreased background activity and clearance of nonspecific blood pool effect [23]. FDS PET imaging showed slightly higher uptake at 120 min in *Aspergillus* infected lungs (0.290 ± 0.030 %ID/mL, *n* = 9) compared with control (0.098 ± 0.008 %ID/mL, *n* = 8) and inflammation (poly(I:C) model) (0.167 ± 0.007 %ID/mL, *n* = 5), although the latter difference was less impressive (Figure 3B,C). With FDG PET, however, high uptake was found in both *Aspergillus* infection and poly(I:C)-induced lung inflammation (Figure 3A,C), although it was higher in the former. To confirm the extent of *Aspergillus* infection, GMS staining was performed. Figure 3D shows an example of the fungal burden in the infected lungs as indicated by the presence of hyphae (stained black). The fungal hyphae were mainly clustered around bronchi/bronchioles consistent with the inoculated conidia having germinated in the lung tissue. Uninfected mice lungs, on the other hand, showed no GMS staining, as expected (Figure 3D). Animals infected with *A. fumigatus* who were found to have no appreciable or minimal GMS staining were excluded from the final analysis. Moreover, animals with rapid progression of disease and secondary kidney failure (acute tubular necrosis related to sepsis) were excluded from the analysis to avoid the effect of blood pooling/increased permeability and poor clearance of the ligand.

### 3.4. PET Imaging of Myositis Models of Infection

**Figure 4:**
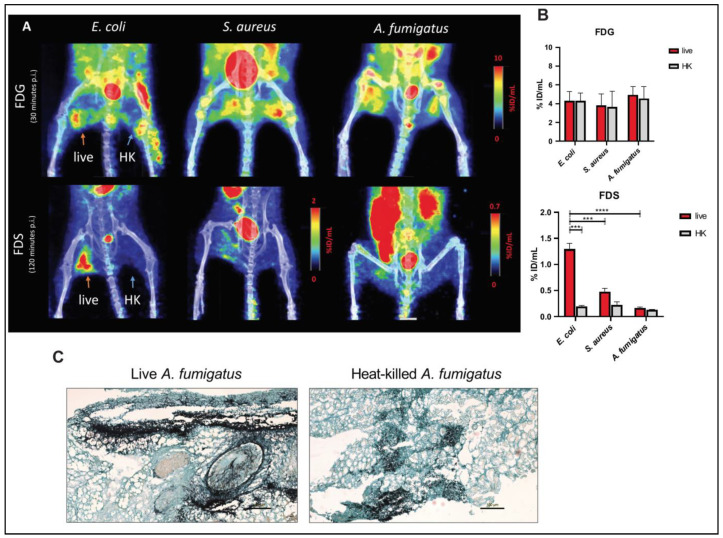
Static ^18^F-fluorodeoxyglucose (FDG) and 2-[^18^F]-fluorodeoxysorbitol (FDS) PET/CT imaging of *E. coli*, *S. aureus,* and *A. fumigatus* myositis models. (**A**) Maximum intensity projections (MIPs) of representative PET/CT images and (**B**) quantified uptake values (%ID/mL) show high FDG uptake in both live (orange arrows) and heat-killed injection sites (blue arrows) in all three models. FDS uptake, on the other hand, is highest in association with live *E. coli* with no appreciable uptake seen on the heat-killed side. *S. aureus* and *A. fumigatus* models show higher FDS uptake in the live versus heat-killed (HK) thighs, however, the uptake associated with live infection is much lower than that seen in live *E. coli* model. (**C**) GMS staining of muscle slices from live and heat-killed (HK) *A. fumigatus* inoculated thighs. Scale bars, 100 µm. Data are expressed as means ± SEM. ***, *p* < 0.001. ****, *p* < 0.0001. (%ID/mL: %Injected dose/mL).

To compare the uptake of FDS in models of fungal and bacterial infections, we used mice that were intramuscularly infected with *A. fumigatus*, *E. coli*, or *S. aureus* and obtained static FDS PET imaging at 120 min. The degree of swelling and lameness as well as the effect on mobility between the fungal and bacterial myositis models were comparable. FDG PET uptake was noted in all animal models, both in the live and heat-killed sides. FDS uptake, on the other hand, was found to be higher in the thigh infected with live *E. coli* (1.294 ± 0.114 %ID/mL, T/NT ratio = 7.053 ± 0.716, *n* = 6) but not with heat-killed *E. coli* (0.193 ± 0.021 %ID/mL, T/NT ratio = 1.040 ± 0.093, *n* = 6). Mild uptake was seen in live *S. aureus* infected thigh, albeit to a lower degree than live *E. coli* (Figure 4A). In the *Aspergillus* myositis model, there was only slightly higher uptake in the live fungus (0.167 ± 0.018 %ID/mL, T/NT ratio = 1.307 ± 0.1535, *n* = 7) compared with the heat-killed inoculated thigh (0.132 ± 0.008 %ID/mL, T/NT ratio = 1.025 ± 0.055, *n* = 7) (Figure 4B and Figure S3). As a comparison, FDG PET accumulated in both thighs in the *Aspergillus* myositis model (live inoculations: 4.935 ± 0.449 %ID/mL, T/NT ratio = 2.885 ± 0.339; heat-killed inoculations: 4.545 ± 0.642 %ID/mL, T/NT ratio = 2.673 ± 0.449, *n* = 4) (Figure 4B and Figure S3). Confirmation of the *A. fumigatus* infection was performed using GMS staining (Figure 4C).

Since FDG and FDS PET were performed at different time points (30 min and 120 min), we performed PET at both 30 and 120 min after injection of FDG in *E. coli* and *S. aureus* myositis models. There were no significant differences in decay-corrected FDG uptake values measured at 30 min and 120 min after injection (1.299 ± 0.190 vs. 1.418 ± 0.205 %ID/mL, *p* = 0.3025, *n* = 6) (Figure S4) confirming the appropriateness of comparison of FDG uptake at 30 min to FDS uptake at 120 min.

To assess the relationship between uptake and size of inoculum, we performed FDS PET imaging on *E. coli* myositis models which were inoculated with 1 × 10^8^ versus 5 × 10^8^ live *E. coli* CFUs in the right thigh. We found that FDS uptake is significantly higher in the 5 × 10^8^ group than in the 1 × 10^8^ group (Figure S5).

### 3.5. PET/CT Imaging of Aspergillus Infection after Intravenous Challenge

To extend our findings to systemic *Aspergillus* infections, we used the intravenous infection model. In this model, the fungal infection is known to spread primarily to the brain and kidneys after inoculation [33,34]. FDS uptake in the brains of infected animals was compared with healthy controls. Representative transverse and coronal PET images of healthy brain and *A. fumigatus* infected brain are shown in Supplementary Figure S6A. The data demonstrated higher uptake in *A. fumigatus*-infected brain (0.187 ± 0.019 %ID/mL, *n* = 5) versus normal brain (0.043 ± 0.002 %ID/mL, *n* = 8) (Figure S6B). GMS staining was performed to confirm the fungal burden in the brain (Figure S6C). We did not perform further evaluation of the BBB integrity in this group, so we cannot fully rule out the possibility of leakage rather than specific uptake of the ligand.

## 4. Discussion

In this study, we found that although there seems to be uptake of ^3^H-sorbitol and FDS by *A. fumigatus* both in vitro and in vivo, this was minimal, with only slightly higher lung uptake in the infected lungs compared with lung inflammation and much lower uptake in fungal myositis compared with *E. coli* myositis.

Invasive Aspergillosis is a life-threatening infectious disease, especially for immunosuppressed patients. Traditional diagnostic methods such as cultures of samples obtained from sputum or BAL can delay the diagnosis and their accuracy can be affected by contamination and antimicrobial initiation. Other tests such as BAL galactomannan for IPA have limited sensitivity and specificity (as low as 69.2% and 72.2%, respectively) [35]. Moreover, invasive biopsies of deep-seated infectious foci risk introducing other infections to immunosuppressed patients and can have serious complications such as pneumothorax or abdominal bleeding. In comparison, molecular imaging (e.g., PET) can potentially provide a non-invasive and fast diagnosis if a fungal-specific ligand can be developed/validated. Our hypothesis in evaluating FDS as a potential ligand for imaging fungal infection was based on previously described sorbitol catabolism pathways in multiple fungi, including *A. fumigatus*. If proven to be an appropriate imaging ligand for fungal infection, the ease of synthesizing FDS from FDG would make it a translatable approach, although one issue would always be how to differentiate *Enterobacterales* infection from fungal infections. The latter scenario, however, is less likely considering the different typical clinical presentations of those infections.

Starting with in vitro assays, our first hurdle was to establish a method to compare fungal growth (namely *A. fumigatus*) with bacterial growth for quantification purposes. While it is relatively easy to normalize bacterial growth to CFUs, this is not as easily done with molds and can underestimate the actual fungal burden [36]. Other approaches to quantifying mold infection include the conidium count of the initial inoculum or quantitative PCR which was described to successfully measure *A. fumigatus* burden in animal models of infection over time [37]. Those approaches, however, would still have limitations as far as directly comparing fungal with bacterial burden considering growth in vitro of both types of pathogens is inherently physiologically different and strongly depends on the conditions of incubation such as growth media and length of incubation, while growth in vivo depends on the immune system status, location of the infectious lesions, and duration of infection.

While fully aware of the limitations of the comparison, we started by comparing the in vitro uptake of ^3^H-sorbitol between *A. fumigatus* and various bacterial strains, namely, *E. coli*, *S aureus*, and *P. aeruginosa*, using weight for normalization purposes. We found that ^3^H-sorbitol uptake was considerably higher in *E. coli* compared with the rest of the evaluated pathogens including *A. fumigatus* (Figure 1). However, since weight is not an optimal normalization approach, we also compared the uptake of ^3^H-sorbitol with that of ^3^H-2-DG in all the assays and measured the ratio of uptake of ^3^H-sorbitol to ^3^H-2-DG. We felt this would provide a good appreciation of the relative preference for sorbitol compared with glucose since the latter is a universal source of energy for pathogens [38]. Using this approach, *E. coli* preference for ^3^H-sorbitol was much higher than all other pathogens with a mean ratio value of 12.7 ± 0.51 at 120 min compared with the mean ratio of 1.2 ± 0.37 for *A. fumigatus* and 1.14 ± 0.29 for *P. aeruginosa*. The rest of the evaluated pathogens had lower ratios consistent with approximately equal or higher preference of glucose to sorbitol. Macrophage cell lines, as expected, showed the least preference for sorbitol compared with 2-DG consistent with minimal, if any, uptake of sorbitol. That would explain the lack of appreciable uptake of FDS in inflammatory noninfectious etiologies [23].

We found similar results using in vitro uptake assays for FDS and FDG with much higher uptake of FDS by *E. coli* when compared with *A. fumigatus*. There was still uptake of FDS by live *A. fumigatus* compared with heat-killed pathogens. FDG uptake on the other hand was high in both *E. coli* and *A. fumigatus*, especially when compared with heat-killed cultures (Figure 1C,D).

Of note is that for all the mold uptake assays (*A. fumigatus* and *A. niger*), we used the hyphal stage of growth to evaluate uptake rather than conidia. That is because the hyphal stage of growth underlies disease manifestations in patients and because we expected very low uptake in conidia considering their dormant nature prior to germination. In order to confirm these distinct patterns of uptake, we performed separate uptake experiments using conidia as well as hyphal cultures that were allowed to grow overnight. We then normalized the in vitro uptake levels of ^3^H-2-DG and ^3^H-sorbitol in hyphal cultures and conidia to the conidium count (10^7^). As expected, we found that the uptake in the hyphae was significantly higher than that in ungerminated conidia (Figure S1) even though the latter were allowed to grow for two hours (the duration of the assay).

Subsequently, we pursued evaluation of FDS uptake in multiple animal models of relevant fungal infections. In order to account for the potential for ligand accumulation in inflammation, associated with increased permeability of the endothelial cells related to cytokine production [39], we developed a lung inflammation model induced by poly(I:C), a synthetic double-stranded RNA commonly used to simulate viral infection [40]. The effects of poly(I:C) on the lungs were confirmed with CT findings of bilateral lung infiltrates and signs of respiratory distress in the animals. Using dynamic imaging, we found higher FDS uptake with slower clearance of FDS in the *A. fumigatus*-infected lungs compared with controls (Figure 2B). The residual uptake at 45 min following injection as well as the uptake at 120 min following injection were also low. This was in contrast to FDG uptake which was much higher in the infected lungs compared with control lungs. Static imaging in the lung inflammation model showed mild FDS retention of the ligand at 120 min, with uptake values between those of control and infected animals (Figure 3). Of note is that we documented GMS staining in all the infected lungs and excluded cases where the fungal load was low or minimal. We also excluded two cases where the animals were very sick with secondary kidney failure and prolonged retention of the ligand in the blood (no or minimal radioactivity in the urinary bladder) since in those cases the appropriate clearance of the ligand is hampered by presumed acute tubular necrosis emblematic of sepsis and shock.

In a second set of experiments meant to compare fungal and bacterial in vivo uptake, we performed FDS and FDG PET imaging in murine myositis models of *E. coli*, *S. aureus*, and *A. fumigatus*. While FDS specifically accumulated in live *E. coli* but not in heat-killed *E. coli* injection sites, as expected, FDG PET could not distinguish live bacteria from heat-killed bacteria (Figure 4A). The FDS uptake in live *Aspergillus* myositis was slightly higher than in the heat-killed inoculation site, however, the T/NT ratio was much lower than that of *E. coli*. This low uptake was noted despite appreciable swelling of the thighs and strong GMS staining (Figure 4B,C).

We believe the low uptake of FDS in *Aspergillus* infection despite known sorbitol metabolism is related to the different uptake mechanisms between bacteria and fungi. In *E. coli*, D-sorbitol is transported inside the bacteria and phosphorylated by a phosphoenolpyruvate-dependent carbohydrate phosphotransferase system (PTS) [21]. Sorbitol-6-phosphate dehydrogenase (encoded by *srlD*) would then convert the substrates into fructose-6-phosphate. In *Aspergillus*, on the other hand, sorbitol dehydrogenase (encoded by *SdhA*) has to be induced first by growth on sorbitol. Once induced, it would directly oxidize D-sorbitol to D-fructose [27]. The expression of *SdhA* is thus dependent on growth media/conditions and is about 20 folds higher when *Aspergillus* is grown on sorbitol-rich media compared with regular media [27] and 14 folds higher compared with sucrose-rich media [41]. This difference in uptake mechanism could explain the lack of uptake of sorbitol and FDS in vitro and in vivo, likely because of low levels of *SdhA* induction. Further confirming this hypothesis, we found a marked increase (23-fold) in ^3^H-sorbitol uptake by *Aspergillus* in vitro when the fungus was grown in sorbitol-rich media compared with regular media prior to incubation with ^3^H-sorbitol (Figure S2). Given that FDS is injected in trace doses, and the duration of uptake is two hours, we do not expect FDS to induce enough *SdhA* expression to increase sorbitol/FDS uptake in vivo. Although *A. fumigatus* has a slight preference for sorbitol over glucose (Figure 1), the availability of the latter at much higher levels in the circulation would probably attenuate the fungus uptake of FDS. This is in contrast to *E. coli* which does not require induction for sorbitol uptake to happen.

In the systemic *A. fumigatus* infection model, we noticed higher FDS uptake in the brains of the infected mice when compared with healthy animals. Infection with *A. fumigatus* was confirmed by GMS staining. Brain fungal infection, however, results in blood–brain barrier disruption and we believe the uptake we are seeing reflects non-specific ligand accumulation due to leakage into the extracellular space at the site of infection rather than uptake by the fungus. Therefore, further studies need to be done to determine the specificity of FDS uptake in brain fungal infections.

The main limitation of our study is the inability to directly compare the fungal hyphal burden to bacterial burden due to differences in growth pattern, dynamics, and metabolism of the pathogens. This is why we utilized weight as a normalizing factor in vitro and also used ^3^H-2-DG at the same time as ^3^H-sorbitol to provide an approximate comparison between glucose and sorbitol metabolism of the various pathogens. Another issue is that we noticed higher uptake of FDS in *S. aureus* myositis compared with *Aspergillus* myositis, which could be related to the difference in growth and uptake mechanisms between the growth media and physiologic environment, especially regarding glucose content. Higher glucose in the blood compared with our growth media (2 g/mL) could explain lower usage of FDS by *Aspergillus* compared with in vitro uptake of sorbitol. Another limitation is that static FDS PET scans were performed 120 min after injection to reduce the background signal, while FDG PET scans were performed 30 min after injection. However, in a subset of animals, we were able to confirm no significant difference of FDG uptake when corrected for decay at 120 min as compared with 30 min after injection (Figure S4).

Identifying fungal-specific noninvasive imaging biomarkers is becoming more of a necessity with the increasing rates of fungal infections in immunocompromised populations, mainly related to improved treatment responses of various malignancies and prolonged survival of transplant patients. Potential imaging biomarkers that target fungi could include various sugars (mono-, di-, and oligosaccharides) that would be specifically used by fungi [42] but not by bacteria or activated immune cells as well as antibodies and antibody fragments that target fungal cell wall components or secreted antigens. Yet, fewer imaging groups are attempting this approach compared with bacterial imaging, likely due to the inherent difficulties in handling fungi compared with bacteria as well as the differences in growth characteristics and metabolism between the species, especially when molds (such as *A. fumigatus*) are targeted. As a result, more enthusiasm is needed to identify different metabolism targets or antibodies/peptides that are specific to fungal infection with appropriate high uptake by the fungus and retention compared with background.

## 5. Conclusions

High selectivity (100 to 2000 times) of a certain PET or SPECT ligand for the targeted pathogen, favoring accumulation in the infectious focus over the background signal in normal tissues, is required for an imaging tracer to adequately visualize infection [13]. In our hands, we found low selectivity for FDS in *A. fumigatus*. Although *A. fumigatus* can accumulate FDS to a limited extent, the uptake is very low both in vitro and in vivo, and therefore, FDS is not a viable ligand for PET imaging of *A. fumigatus* infections. At this point, FDS remains specific for Gram-negative *Enterobacterales* infections.

## Data Availability

Data reported in this paper is contained within the article and Supplementary Material.

## Supplementary Materials

The following are available online at https://www.mdpi.com/article/10.3390/jof8010025/s1, Figure S1: In vitro uptake of ^3^H-sorbitol and ^3^H-2-DG in *A. fumigatus* cultures at spore versus hyphal stages of growth. Figure S2: In vitro uptake of ^3^H-sorbitol by *A. fumigatus* on sorbitol-rich culture media or regular media. Figure S3: Quantification of static FDG and FDS PET imaging of *E. coli*, *S. aureus,* and *A. fumigatus* myositis models. Figure S4: Static PET/CT imaging with FDG in bacterial myositis model. Figure S5: Static PET/CT imaging with FDS in myositis model induced with different *E. coli* inocula (1 × 10^8^ or 5 × 10^8^). Figure S6: Static PET/CT imaging with FDS in peripheral infected model.
