# Supplementary material for: Evaluation of 2-[18F]-Fluorodeoxysorbitol PET Imaging in Preclinical Models of Aspergillus Infection
Source: J Fungi (Basel). 2021 Dec 28;8(1):25. doi: 10.3390/jof8010025 (PMC8780649; doi:10.3390/jof8010025)
Supplement: Supplementary file 1 [file jof-08-00025-s001.zip › jof-1520180-supplementary.pdf]

Journal of
Fungi

MDPI

Supplemental figures:

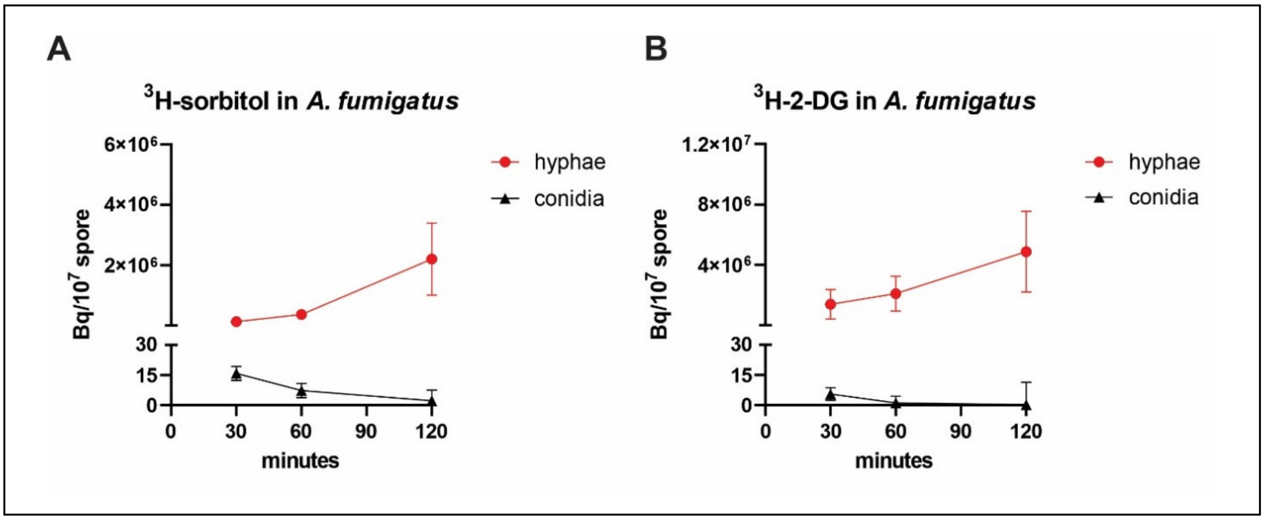

Figure S1. *In vitro* uptake of ^{3}H-sorbitol and ^{3}H-2-Deoxyglucose (DG) in *A. fumigatus* cultures at conidial versus hyphal stages of growth. Uptake in the hyphal stage is much higher than uptake at conidial/early germination stage.

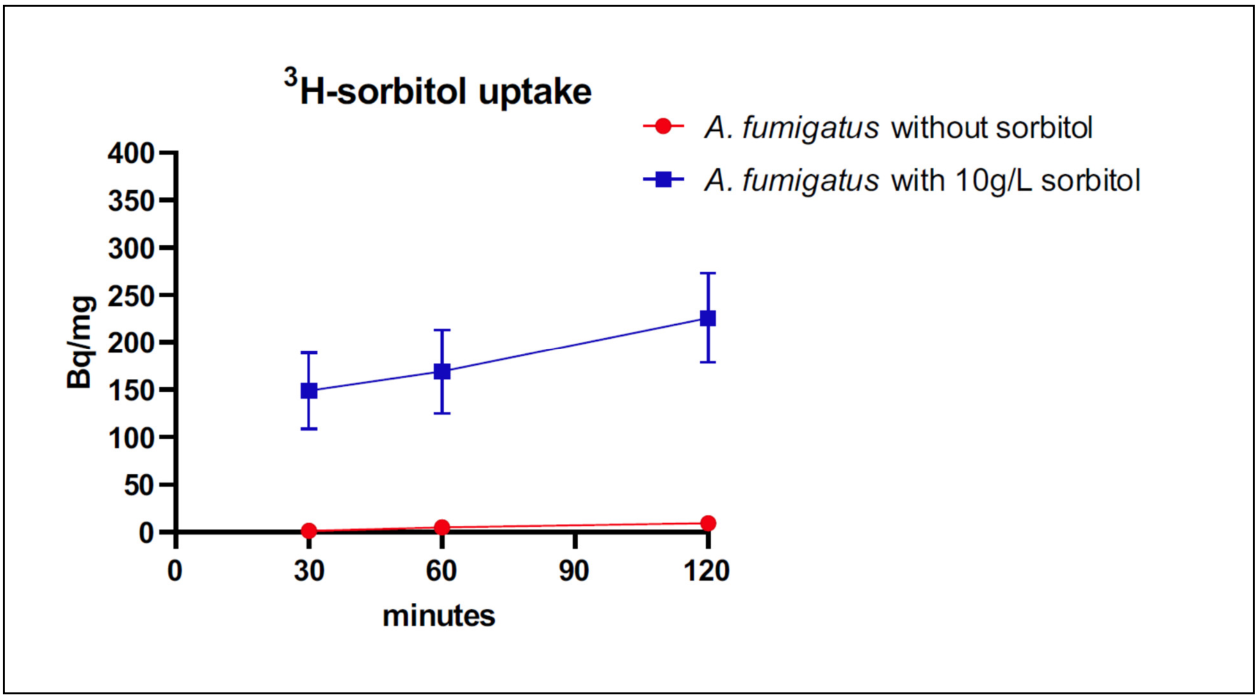

Figure S2. *In vitro* uptake of ^{3}H-sorbitol by *A. fumigatus* is markedly increased (~23 fold at 120 minutes) when the fungus is grown on sorbitol-rich culture media compared to regular (no sorbitol added) media.

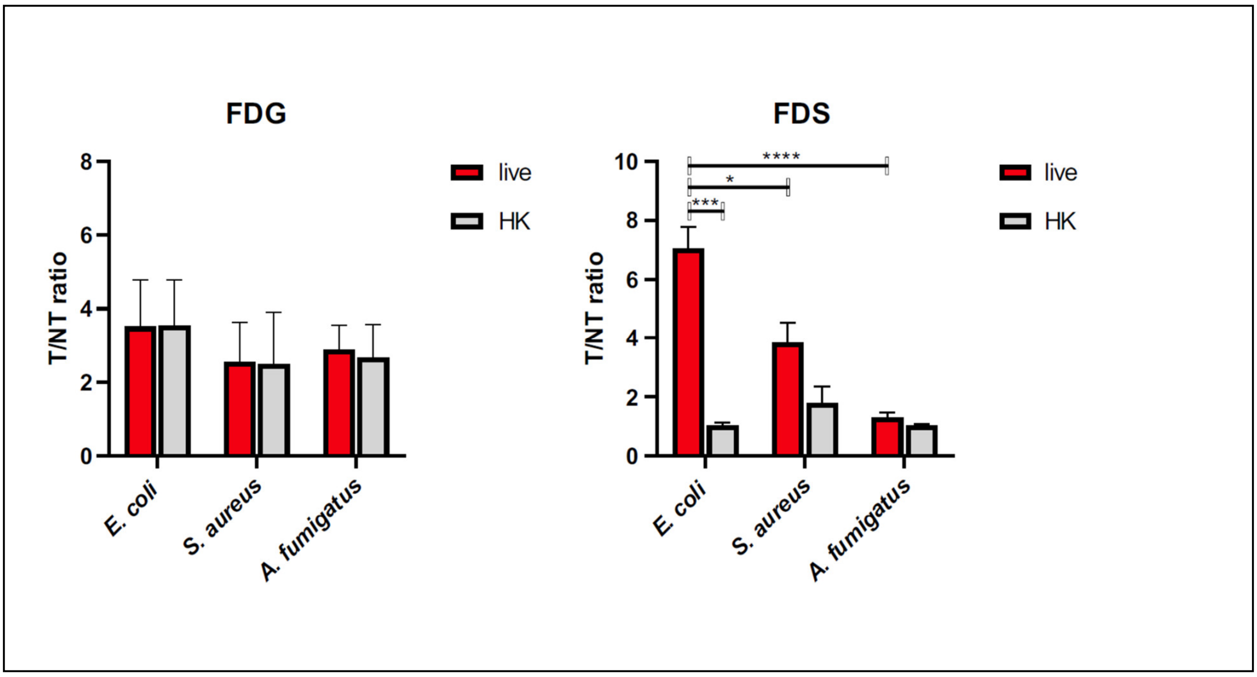

Figure S3. Quantification of static ^{18}F-fluorodeoxyglucose (FDG) and 2-[^{18}F]-fluorodeoxysorbitol (FDS) PET imaging of *E. coli*, *S. aureus* and *A. fumigatus* myositis models. Values are shown as target to non-target (T/NT) ratio. Data are expressed as means ± SEM. *, $p < .05$. ***, $p < .001$. ****, $p < .0001$.

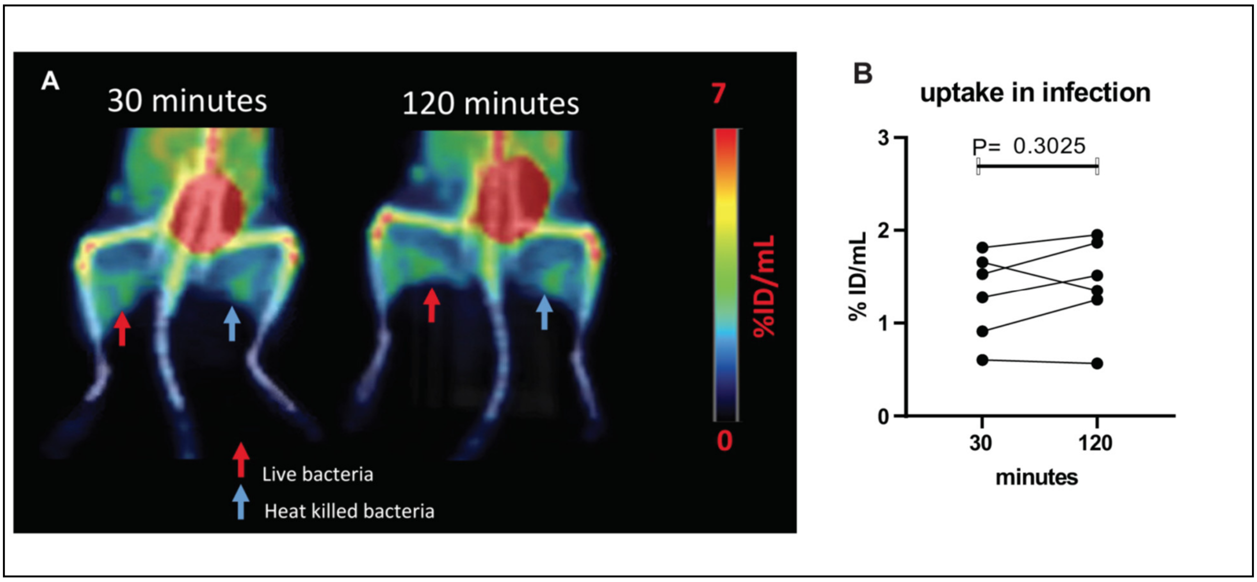

Figure S4. Static PET/CT imaging with ^{18}F-fluorodeoxyglucose (FDG) in bacterial myositis model. A. Representative PET/CT images obtained at 30 or 120 minutes after injection of FDG. Live and heat-killed bacteria inoculated thighs are indicated by red and blue arrows respectively. B. No significant differences were seen in FDG uptake values (corrected for decay) between 30 and 120 minutes after injection ($n = 6$).

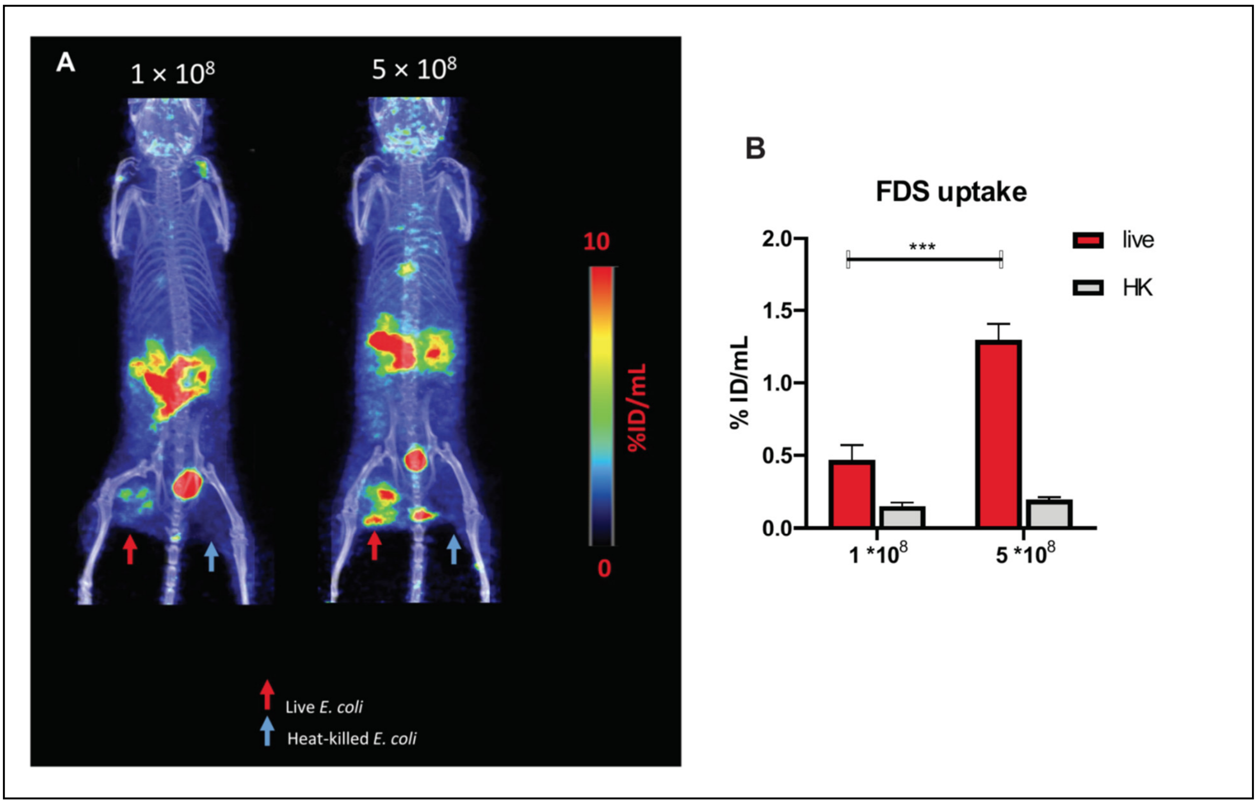

Figure S5. Static PET/CT imaging with 2-[^{18}F]-fluorodeoxysorbitol (FDS) in myositis model induced with different *E. coli* inocula (1×10^8 or 5×10^8). A. Representative PET/CT images obtained at 120 minutes after injection of FDS show higher uptake with the higher dose of inoculum. Live and heat-killed E. coli inoculated thighs are indicated by red and blue arrows respectively. B. quantified uptake values (% Injected Dose/mL) also show higher uptake with the higher dose of inoculum ($n = 6$). Data are expressed as means ± SEM. ***, $p < .001$.

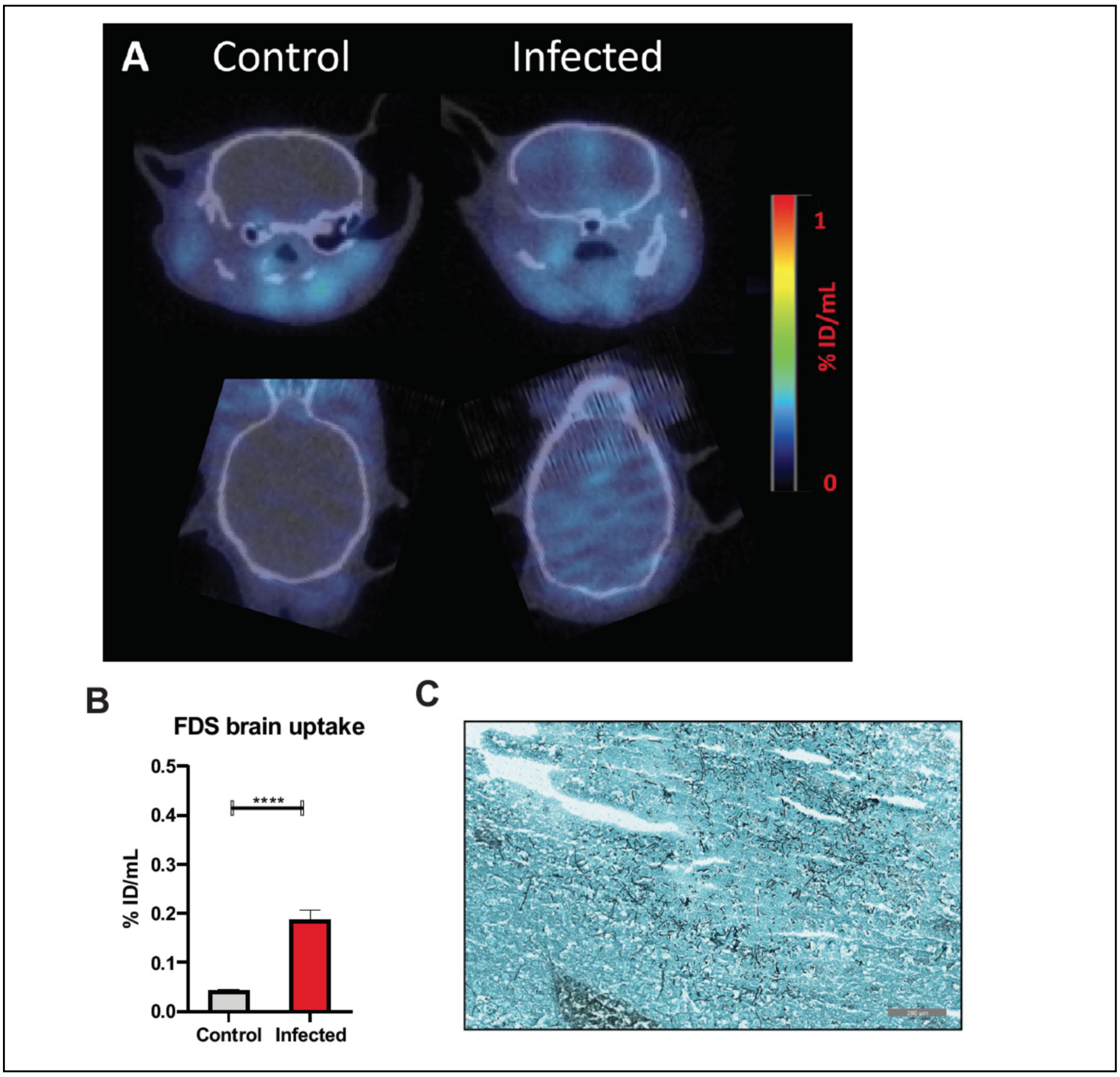

Figure S6. Static PET/CT imaging with 2-[^{18}F]-fluorodeoxysorbitol (FDS) in peripheral infected model. A. Representative PET/CT images of FDS 120 minutes post injection in control or intravenous *A. fumigatus* infected mice. Transverse (top) and coronal (bottom) slices of brain PET/CT images are shown. B. Quantified uptake of FDS in the brain of control or Aspergillus intravenous infected mice at 120 minutes after injection (n = 5). C. GMS staining of brain slices from an *A. fumigatus* infected mouse on day 3 after infection. Scale bars 200 μm. Data are expressed as means ± SEM. ****, $p < .0001$.
